# A qualitative study on emergency department nurses' experiences of coping with moral distress based on self-efficacy theory

**DOI:** 10.3389/fpubh.2026.1849971

**Published:** 2026-07-03

**Authors:** Linqi Gan, Liqun Huang, Shirong Wu, Huanmei Li, Zhenhui Mao, Qiuying Deng

**Affiliations:** 1 The Second Clinical College of Guangzhou University of Chinese Medicine, Guangzhou, China; 2 Emergency Department, The Second Affiliated Hospital of Guangzhou University of Chinese Medicine, Guangzhou, China; 3 School of Nursing, The Hong Kong Polytechnic University, Hong Kong, Hong Kong SAR, China

**Keywords:** coping ability, emergency department nurses, moral distress, qualitative research, self-efficacy

## Abstract

**Objective:**

Grounded in Bandura's self-efficacy theory, this study aims to identify the key factors influencing how emergency department (ED) nurses cope with moral distress, providing an evidence base for targeted interventions to enhance their coping capacity and psychological well-being.

**Methods:**

A qualitative descriptive design was adopted. Between November and December 2025, 17 ED nurses from a tertiary general hospital were recruited through purposive sampling with maximum variation. One-to-one semi-structured interviews were conducted and analyzed using thematic framework analysis. To enhance trustworthiness, the data were independently coded by two researchers, and peer debriefing and an audit trail were employed.

**Results:**

Four main themes and 10 subthemes emerged from the analysis. ED nurses' coping self-efficacy is influenced by both personal confidence and the social context surrounding ethical decision-making. Participants described recurrent tensions between institutional rules and humanitarian care, triage dilemmas arising from staff and equipment shortages, and defensive practice following complaints, disciplinary action, or workplace violence. Self-efficacy was further influenced by the availability of collegial discussion, senior guidance, and managerial recognition, as well as by a perceived lack of empathy among peers. Nurses also reported that disgust, emotional exhaustion, and value conflict undermined their willingness and confidence to sustain care, whereas indirect exposure to colleagues' traumatic experiences reinforced self-protective boundaries. The most frequently reported subtheme was value conflict and emotional exhaustion (76.5%), followed by conflict between institutional mandates and humanitarian care, and triage dilemmas under resource scarcity.

**Conclusion:**

ED nurses' coping self-efficacy in the face of moral distress is socially situated and dynamically shaped by institutional constraints, resource scarcity, interpersonal interactions, emotional arousal, and exposure to traumatic clinical events. Interventions should move beyond individual-level coping training to address the organizational, relational, and ethical conditions of emergency care through context-sensitive support, mentorship, ethics training, and psychologically informed, team-based strategies.

## 1 Introduction

The emergency department (ED) is universally recognized as one of the most high-pressure and ethically challenging settings within healthcare systems, responsible for the rapid triage and management of a substantial volume of critically ill, injured, and unstable patients. As frontline healthcare providers, ED nurses face uniquely multifaceted pressures that extend beyond the technical demands of emergency care: in addition to mastering time-sensitive clinical procedures (1) (e.g., cardiopulmonary resuscitation, trauma stabilization), they frequently encounter complex moral decision-making scenarios requiring navigation of conflicting ethical principles, legal obligations, interpersonal dynamics, and resource constraints (2). Moral distress, conceptualized by Jameton (3) as the psychological anguish arising when an individual knows the morally correct course of action but is hindered by external or internal barriers from implementing it, is particularly pervasive among ED nurses due to the acute, unpredictable nature of their work environment (4). In China, existing studies suggest that ED nurses experience moderate-to-high levels of moral distress (5–7).

Extensive empirical evidence has documented the detrimental impacts of persistent moral distress on ED nurses' wellbeing and professional practice. Studies indicate that chronic moral distress is strongly associated with adverse psychological outcomes such as anxiety, depression, compassion fatigue, and burnout (8–10), as well as reduced job satisfaction, eroded professional identity, and increased turnover intentions (11). Beyond individual consequences, unaddressed moral distress can compromise the quality of patient care—including reduced attention to detail, diminished empathy, and suboptimal clinical decision-making—and undermine the ethical integrity of healthcare organizations (12). A recent qualitative systematic review further synthesized the multidimensional drivers of moral distress in ED nurses and called for theory-driven qualitative inquiry into how nurses actively cope with such experiences (13). Given these far-reaching implications, understanding how ED nurses cope with moral distress and identifying modifiable factors that enhance their coping effectiveness is a critical priority for nursing research and practice.

Proposed by Bandura (14), self-efficacy is a domain-specific belief that fosters resilience and proactive coping. While recognized as a protective factor against moral distress in nursing, its role in ED settings is under-explored. Current quantitative evidence lacks depth regarding subjective coping mechanisms, necessitating qualitative research to elucidate how self-efficacy shapes ED nurses' lived experiences of moral distress.

A recent study has predominantly documented the sources, manifestations and emotional consequences of moral distress, while paying limited attention to how nurses actively cope with such experiences (13). Most prior qualitative work has been descriptive in nature and has not been explicitly anchored in a theoretical framework, which constrains its capacity to explain how coping confidence is formed, eroded or reconstructed. In particular, the four hypothesized sources of self-efficacy proposed by Bandura (14)—direct (mastery) experience, verbal persuasion and social support, physiological and emotional arousal, and vicarious experience—have rarely been used as an integrative lens to interpret ED nurses' lived experiences of moral distress. Evidence from high-pressure tertiary EDs in China, where institutional pressures, workplace violence and family-driven decision conflicts are pronounced, also remains scarce. Against this background, the present study contributes a theory-driven, context-specific qualitative account that complements and extends previous descriptive work by mapping ED nurses' coping experiences onto Bandura's four sources of self-efficacy. The present study therefore seeks to address this gap by examining how the four sources of self-efficacy operate in ED nurses' coping with moral distress, and by providing context-specific implications for ethics-oriented support in emergency nursing.

Therefore, drawing upon Bandura's Self-Efficacy Theory as a conceptual framework, this qualitative study aims to address the current literature gap by exploring the subjective experiences of ED nurses. The primary objective is to understand how these nurses conceptualize moral distress and to uncover the underlying processes through which self-efficacy beliefs mobilize their coping strategies and resilience in a high-acuity setting.

## 2 Methods

### 2.1 Study design

This study employed a qualitative descriptive design using semi-structured interviews and thematic framework analysis (15). This design is well suited to examining the subjective coping experiences of ED nurses confronting moral distress, as it facilitates a rich, contextualized description of participants' perspectives while remaining close to their own accounts. Bandura's self-efficacy theory served as the a priori conceptual framework guiding the development of the interview guide, coding, and theme interpretation, thereby supporting an in-depth analysis of the psychological processes, behavioral strategies, and contextual factors shaping nurses' responses. All procedures for data collection, analysis, and reporting were conducted in accordance with the Consolidated Criteria for Reporting Qualitative Research (COREQ-32) guidelines. The completed COREQ-32 checklist is provided as Supplementary Material 1 to ensure transparency in reporting.

### 2.2 Participants

According to the principle of maximum variation, purposive sampling was conducted from November to December 2025 in a tertiary general hospital, selecting ED nurses who had experienced moral distress. Inclusion criteria: (1) registered nurses employed in ED; (2) working in ED for at least 1 year; (3) having experienced moral distress; (4) voluntarily participating. Exclusion criteria: (1) nurses on rotation or further training; (2) nurses on leave for maternity, lactation, illness, or study outside the hospital. Data collection and analysis were conducted concurrently. Thematic saturation was operationalized as the point at which no new codes or subthemes emerged during ongoing line-by-line coding by two independent researchers. After the 15th interview, both coders agreed that no new conceptual content was being generated; two additional interviews (N16 and N17) were then conducted to confirm thematic redundancy, after which recruitment ceased. Seventeen nurses were interviewed and coded as N1–N17. Two eligible nurses declined participation because of scheduling constraints; no participants withdrew after consent. To reflect the industry's gender composition, five male nurses (29.4%) were included to explore gender differences in moral dilemma coping experience.

### 2.3 Research team and reflexivity

The research team consisted of clinical nursing experts with emergency experience and a foundation in qualitative research, including nursing specialists and two researchers with postgraduate-level nursing training. The interviews were conducted by Linqi Gan and Liqun Huang. At the time of the study, Linqi Gan was a postgraduate nursing student, and Liqun Huang was a clinical nurse with postgraduate training in nursing. Both interviewers were female. Both had received prior training in qualitative interviewing techniques and were familiar with the clinical context of emergency care, which facilitated rapport-building and contextual understanding during data collection. No prior personal relationship was established with participants before study commencement.

### 2.4 Procedure

Before the interviews, participants were informed of the study purpose, confidentiality procedures, and the researchers' professional background and research interest in ED nurses' experiences of moral distress. The semi-structured interview guide was developed through an iterative process involving a structured literature review, team discussions, and explicit mapping to Bandura's self-efficacy theory. Two pilot interviews were conducted with eligible ED nurses to evaluate question clarity and sequencing, leading to minor wording refinements; these pilot data were excluded from the final analysis. The finalized guide comprised seven open-ended core questions designed to elicit participants' conceptualizations of moral distress, lived experiences, perceived impacts, coping strategies, facilitators of coping, and perceived support needs (Table 1). Subsequently, two researchers from the team conducted one-on-one, audio-recorded interviews in a quiet room within the hospital, lasting between 20 and 50 min. No non-participants were present during the interviews. Prior to each session, the study's purpose, confidentiality, and recording procedures were explained to obtain informed consent, and appropriate probing techniques were utilized during the interviews to ensure comprehensive data collection.

**Table 1:** Core interview questions and their alignment with Bandura's self-efficacy framework.

No	Core question	Theoretical focus
1	Could you share your understanding of moral distress?	Cognitive appraisal of the stressor
2	Could you describe specific experiences of moral distress you have encountered while working in the ED?	Mastery experiences
3	How do you perceive the impact of these experiences on you?	Physiological and affective states
4	How do you cope with these situations?	Behavioral coping strategies
5	What factors do you think have helped you cope with these dilemmas?	Sources of efficacy (vicarious experience, verbal persuasion, social support)
6	What additional support do you feel you need during this process, and why?	Contextual and organizational facilitators
7	Is there anything else you would like to share?	Open-ended closure

### 2.5 Quality Control

Quality control was maintained throughout the study. Participant recruitment strictly followed the inclusion and exclusion criteria, and all participants provided informed consent before voluntary participation. Interviews were audio-recorded and transcribed verbatim within 24 h. Field notes were taken during and immediately after each interview to capture nonverbal cues and contextual information. Data were checked to ensure coding accuracy and logical consistency.

Credibility was enhanced through prolonged engagement in the ED, independent dual coding in NVivo 12 Plus, and peer debriefing with a senior qualitative researcher not involved in the study. Dependability and confirmability were supported by an audit trail including recordings, transcripts, and revisions to the coding framework. Transferability was strengthened by providing detailed descriptions of participant characteristics (Table 2) and the ED context.

**Table 2:** Basic information of interviewees (*n* = 17).

No	Gender	Age	Education	Professional title	Working years (Total)	Working years in ED
N1	Female	29	Bachelor	Nurse	7	6
N2	Female	39	Bachelor	Supervisor nurse	18	17
N3	Female	45	Bachelor	Supervisor nurse	22	20
N4	Female	38	College	Supervisor nurse	7	5
N5	Female	23	Bachelor	Nurse	2	1
N6	Male	39	Bachelor	Supervisor nurse	15	14
N7	Male	32	Bachelor	Supervisor nurse	10	8
N8	Female	32	Bachelor	Supervisor nurse	10	9
N9	Female	29	Bachelor	Nurse	7	5
N10	Male	25	College	Nurse	3	1
N11	Female	23	College	Nurse	2	1
N12	Female	34	College	Nurse	8	7
N13	Female	28	Bachelor	Nurse	6	4
N14	Female	27	Bachelor	Nurse	7	5
N15	Male	39	Bachelor	Supervisor nurse	13	13
N16	Female	26	Bachelor	Nurse	2	2
N17	Male	41	College	Nurse	15	15

To reduce interpretive bias, both coders documented their professional backgrounds and prior assumptions about moral distress and self-efficacy throughout the analysis. Formal member checking of transcripts was not feasible because of participants' rotating shifts and clinical workload; instead, key interpretations were summarized at the end of each interview to confirm understanding. This limitation is acknowledged.

### 2.6 Data analysis

Two researchers transcribed the interviews within 24 hours of completion. NVivo 12 Plus software was used to facilitate coding and theme development. Interview transcripts were analyzed using the thematic framework method (15, 16), with Bandura's four sources of self-efficacy—mastery experiences, vicarious experiences, verbal persuasion, and physiological and affective states—serving as the a priori analytical framework. Two coders read the transcripts repeatedly to familiarize themselves with the data, identified meaning units relevant to nurses' moral distress and self-efficacy, and developed a preliminary coding framework on this basis. The two coders then independently coded the data using this framework, while data that did not fit the predefined categories were analyzed inductively to generate additional subthemes. Coding discrepancies were resolved through discussion with reference to the original transcripts until consensus was reached. The final themes and subthemes were refined by integrating the theoretical framework with participants' lived experiences, and representative quotations were selected to present the findings.

## 3 Results

Based on Bandura's self-efficacy theory, four themes and 10 subthemes were identified from the interview data, reflecting how emergency nurses perceived and reconstructed their confidence in coping with morally distressing situations. These themes corresponded to four major sources of self-efficacy: direct experience, external support and verbal persuasion, physiological and emotional arousal, and vicarious experience. To highlight the relative salience of the findings, the frequency and proportion of each subtheme are presented in Table 3.

**Table 3:** Themes, subthemes and number of participants who spontaneously mentioned each subtheme (*n* = 17).

Theme	Subtheme	*n*	%
Theme 1: Reshaping Self-Efficacy Through Direct Experience	Conflict Between Institutional Mandates and Humanitarian Care	12	70.6
	Triage Dilemmas Under Resource Scarcity	10	58.8
	Feedback-Driven Shifts From Confidence to Defensive Practice	8	47.1
Theme 2: Strengthening Self-Efficacy Through External Support and Verbal Persuasion	Access to Collegial and Interdisciplinary Support	9	52.9
	Mentorship, Recognition, and Ethics-Oriented Guidance	8	47.1
	Peer Insensitivity as a Source of Additional Distress	4	23.5
Theme 3: Physiological and Emotional Arousal in Coping Self-Efficacy	Professional Responsibility Amid Physiological Aversion	9	52.9
	Value Conflict and Emotional Exhaustion	13	76.5
Theme 4: Reconstructing Coping Confidence Through Vicarious Experience	Peer Experience Sharing as a Source of Coping Confidence	8	47.1
	Indirect Trauma and the Redefinition of Professional Boundaries	5	29.4

*n* = number of participants spontaneously mentioning the subtheme; % = proportion of the total sample (*N* = 17). Frequencies denote spontaneous mentions in open-ended interviews, reflecting subtheme salience within the dataset. Subthemes are not mutually exclusive (i.e., a single participant could contribute to multiple subthemes).

### 3.1 Theme 1: Reshaping Self-Efficacy Through Direct Experience

Participants described direct clinical experience as a major source shaping their self-efficacy in ethically challenging situations, consistent with previous studies (1). In particular, the outcomes of their actions and decisions strongly influenced their subsequent confidence, willingness to engage, and perceived professional agency.

#### 3.1.1 Conflict Between Institutional Mandates and Humanitarian Care

Most participants (*n* = 12) reported intense moral distress when institutional rules or procedural requirements prevented them from acting according to their professional judgment and commitment to minimizing unnecessary patient suffering. In such situations, participants often felt unable to act in ways they believed were most respectful or beneficial to the patient.

N12 recalled performing prolonged cardiopulmonary resuscitation on an unidentified patient following suicide:

“*For the patient who jumped from a building, you know this CPR is meaningless, but without the family present, we couldn't stop. Watching him like that, I felt terrible; it felt like disrespecting him.”*

Similarly, N4 described continuing resuscitation under persistent family demands:

“*Pressing for that long, the ribs were fractured and caved in. Letting him go like that felt so uncomfortable and disrespectful to the deceased.”*

These accounts reflected a reduced sense of control when nurses were required to follow institutional or procedural expectations that conflicted with their ethical judgment.

#### 3.1.2 Triage Dilemmas Under Resource Scarcity

Resource shortages created another form of direct ethical challenge. When staff, equipment, or time were insufficient, nurses were required to prioritize among multiple critically ill patients, which often generated anxiety, hesitation, and fear of long-term psychological burden.

N17 described the distress of having only one defibrillator available for two urgent patients:

“*One was an older patient with acute myocardial infarction, and the other was a young man with massive hemorrhage following a car accident... Those few minutes were agonizing. I was terrified of delaying either's treatment. Every second felt like torment. I even thought, if I made the wrong allocation and someone died, would I carry this psychological burden for life?”*

Participants also noted that triage principles could become difficult to implement in chaotic emergency contexts. N3 explained that family members at the scene sometimes disrupted standard prioritization:

“*At the scene, having family members present changes everything. They just drag you onto the ambulance and demand you save their relative first… You are forced to prioritize patients whose families are calling for help, while those who are unconscious are left behind.”*

These experiences appeared to weaken nurses' confidence in making timely and ethically defensible decisions under pressure.

#### 3.1.3 Feedback-Driven Shifts from Confidence to Defensive Practice

Participants reported that the consequences of ethical decision-making often shaped how they approached similar situations in the future. Positive outcomes tended to strengthen confidence, whereas complaints, punishment, or workplace violence often led to defensive coping and reduced engagement.

N13 described how a complaint following her referral advice changed her practice:

“*The biggest impact is that I won't explain too much anymore… Regardless of whether the patient is right or wrong, as long as it doesn't cross the bottom line, we just satisfy them. I follow the rules because the hospital's rules will protect me.”*

For some participants, workplace violence further undermined their sense of professional commitment. N14 recalled being slapped while trying to stop a psychiatric patient from jumping out of a window:

“*I was here to help you, yet you hit me… Other people's lives are worth valuing, and mine is equally valuable.”*

Such experiences suggested a shift from active moral engagement toward self-protective, rule-bound practice (rather than ethically engaged practice).

### 3.2 Theme 2: Strengthening Self-Efficacy Through External Support and Verbal Persuasion

Participants described external support as an important resource for coping with moral distress. Collegial discussion, senior guidance, managerial recognition, and structured ethics support were all perceived as factors that reduced uncertainty and reinforced professional confidence.

#### 3.2.1 Access to Collegial and Interdisciplinary Support

About half of the participants (*n* = 9) emphasized that timely communication with colleagues or senior staff helped them process difficult decisions and reduce self-blame. Support after emotionally challenging cases was described as especially important.

N17 highlighted the value of post-shift conversations:

“*After work, chatting with colleagues… discussing if what we did met medical standards helps prevent self-blame and relieves anxiety. It helps you face your emotions rationally and avoid getting stuck in rumination.”*

N8 illustrated how a senior colleague's verbal reassurance directly altered her subsequent coping behavior after a contested resuscitation decision:

“*My head nurse went over the whole case with me and said, ‘Given the information you had at that moment, your decision was reasonable.' After that, I stopped second-guessing every order I gave and was able to act decisively in the next similar case instead of freezing.”*

These accounts suggested that accessible peer discussion functioned as a practical and emotional buffer following ethically difficult encounters, and that explicit verbal affirmation from trusted senior colleagues could shift nurses from hesitant, ruminative coping toward more confident, action-oriented practice.

#### 3.2.2 Mentorship, Recognition, and Ethics-Oriented Guidance

Participants also emphasized the importance of mentorship and positive feedback, particularly for novice nurses. Senior nurses were seen as playing a key role in guiding less experienced staff through ethically charged situations and helping them develop confidence.

N15 noted the need for psychologically informed support for younger nurses:

“*Support for new nurses should focus on psychological development. […] Often, they may not have the same level of emotional maturity as those of us who have been working for many years. […] As the older generation of nurses, we should try to help them adjust their mindset from a psychological perspective.”*

In addition, participants suggested that ethics-related experience sharing and simulation-based training could strengthen preparedness. N17 proposed:

“*There could be regular ethics training sessions where experts in ethics or experienced senior nurses can explain the methods for dealing with such situations.”*

She further added:

“*Simulated scenarios could be used for practical exercises […] Some psychological support measures can be taken, such as group mutual assistance, mutual encouragement, and there are also some online and offline courses specifically designed to address moral dilemmas.”*

These responses indicated that structured guidance and affirming feedback were perceived as important in building coping confidence.

#### 3.2.3 Peer Insensitivity as a Source of Additional Distress

In contrast, a lack of empathy from others, especially those within the profession, was described as aggravating distress. Participants reported that when their difficulties were dismissed or normalized in a harsh manner, they felt more isolated and emotionally burdened.

N13 described an encounter with a retired senior nurse as a patient:

“*She told me, ‘Don't be impatient; everything you're doing is what you're supposed to do. We all went through this.' She clearly knows what our profession endures, yet she used that to make things difficult for the next generation. You can't scold her to her face; the anger just gets stuck in your throat. That was the most agonizing part.”*

Such experiences suggested that the absence of professional empathy could undermine emotional support and intensify moral distress.

### 3.3 Theme 3: Physiological and Emotional Arousal in Coping Self-Efficacy

Participants described their physiological reactions and emotional states as closely linked to their perceived ability to continue functioning effectively in ethically difficult situations. Feelings such as disgust, exhaustion, hesitation, and internal conflict often affected both their emotional regulation and their confidence in sustaining care. Within this dataset, we use the term moral numbness to refer to a self-protective dampening of emotional and ethical responsiveness that participants reported after prolonged or repeated exposure to morally distressing events; it is characterized by reduced affective engagement, mechanical task completion, and a sense of inner detachment, rather than by indifference toward patients.

#### 3.3.1 Professional Responsibility Amid Physiological Aversion

Over half of the participants (*n* = 9) described situations in which they had to suppress strong bodily discomfort in order to provide necessary care. Although they continued to perform their duties, these moments revealed the strain involved in maintaining professional responsibility under emotionally and physically aversive conditions.

N14 described caring for an older patient found collapsed in a toilet:

“*His clothes were completely wet, reeking of urine. My stomach churned, and I kept wanting to gag. It felt so dirty, and I didn't want to touch him. But you have no choice… No matter how smelly or dirty, you still have to perform those procedures to keep him alive.”*

This account showed how nurses had to manage immediate physiological reactions while continuing to act in accordance with professional obligations.

#### 3.3.2 Value Conflict and Emotional Exhaustion

Participants also reported that their emotional investment varied across patients and circumstances, sometimes creating internal conflict between personal feelings and professional ideals. These differences were often accompanied by guilt, fatigue, or self-questioning.

N6 reflected on differences in emotional motivation during resuscitation:

“*Resuscitating a 90-year-old versus a 30-year-old brings very different levels of motivation and willingness… Facing a 90-year-old, though I will complete the work, I feel a bit of burnout and less enthusiasm. This difference in starting point causes internal conflict, making me feel I haven't reached that noble ‘benevolence of a healer' level.”*

N2 explicitly described an emerging sense of moral numbness as a protective response to repeated exposure:

“*After so many years, I notice I don't react the way I used to. When another resuscitation fails, I just go through the steps—record, clean up, move to the next bed. It is not that I don't care; it's that if I let myself feel it every time, I wouldn't be able to walk back into this department tomorrow.”*

These narratives indicated that emotional exhaustion and value-based tension could affect nurses' confidence in maintaining consistent ethical commitment across cases.

### 3.4 Theme 4: Reconstructing Coping Confidence Through Vicarious Experience

In addition to firsthand clinical experience, participants described learning from the experiences of others. Observing colleagues, hearing about difficult cases, and witnessing the consequences of workplace violence all contributed to how nurses understood professional boundaries, self-protection, and appropriate coping strategies.

#### 3.4.1 Peer Experience Sharing as a Source of Coping Confidence

Participants reported that experienced colleagues provided not only practical assistance but also a model for interpreting and handling ethically difficult situations. Seeking support from senior nurses was described as a common strategy when facing uncertainty.

N10 stated:

“*When encountering such things, my first reaction is to go to the senior nurses in the department and ask them to get involved and help handle the situation. If there's something that really can't be resolved, I'll then seek help from above—there are the head nurse and the director above me.”*

N5, a junior nurse, described how observing a senior colleague's response to an aggressive family member reshaped her own coping repertoire:

“*I had never seen anyone stay so calm in that situation. She just stepped back, called security, and kept explaining in a steady voice. The next time something similar happened to me, I tried to do the same instead of arguing back, and it actually worked.”*

These accounts suggested that observing and relying on more experienced staff helped participants develop confidence in seeking help and managing complex situations.

#### 3.4.2 Indirect Trauma and the Redefinition of Professional Boundaries

Participants also described how indirect exposure to severe workplace violence shaped their professional outlook. Even when they were not direct victims, such events remained vivid and influenced how they balanced caregiving with self-protection.

N7 recounted a violent incident involving a colleague:

“*A man rushed into the hospital with a knife and started attacking people. A young colleague at the triage desk was severely injured while trying to shield herself… It's been two years, but the image is still vivid.”*

This kind of indirect traumatic exposure appeared to heighten awareness of vulnerability in the emergency setting and contributed to a more cautious understanding of professional responsibility and personal safety.

## 4 Discussion

This study explored the moral distress experiences of ED nurses through the lens of self-efficacy theory. The findings suggest that nurses' responses to moral distress were shaped by four interrelated sources of self-efficacy: direct experience, external support, physiological and emotional arousal, and vicarious experience. Together, these dimensions influenced nurses' confidence in ethical action, decision-making, emotional regulation, and help-seeking in high-pressure clinical contexts. These findings also resonate with a recent qualitative systematic review, which identified appraisal processes and perceived self-efficacy as central to how ED nurses interpret and respond to morally distressing situations (13).

Direct experience emerged as a prominent source shaping self-efficacy. Repeated exposure to futile resuscitation, institutional constraints, and resource scarcity often weakened nurses' perceived agency and reduced confidence in ethically appropriate action. In some cases, negative feedback and disciplinary consequences further reinforced defensive practice, suggesting that unresolved moral distress may gradually shift nurses from patient-centered moral engagement toward rule-oriented self-protection. This finding is consistent with prior research showing that repeated ethically adverse outcomes can erode professional confidence and contribute to withdrawal, burnout, and reduced moral resilience (6).

External support functioned as an important protective factor. Participants emphasized the value of collegial discussion, senior mentorship, and managerial recognition in reducing self-doubt and helping them cognitively reframe ethically difficult events. Conversely, peer indifference or moral pressure intensified distress. These findings indicate that self-efficacy in morally challenging situations is not solely an individual attribute, but is also socially constructed and reinforced within team and organizational environments. Supportive leadership, structured debriefing, and ethics-focused mentorship may therefore be critical for sustaining nurses' coping confidence.

Physiological and emotional arousal also substantially influenced coping efficacy. Participants described disgust, hesitation, emotional exhaustion, and value conflict when caring for certain patients or making high-stakes decisions. These emotional reactions did not necessarily prevent action, but they weakened ED nurses' confidence in carrying out the ethical action they believed was required. This finding highlights that moral distress is not only cognitive or ethical in nature, but also embodied. Interventions that strengthen emotional regulation and reflective capacity may therefore enhance ED nurses' confidence in maintaining humane care under distressing conditions.

Vicarious experience further shaped professional boundary awareness. Learning from senior nurses and observing colleagues' traumatic experiences helped participants reshape their understanding of professional boundaries—when to persist, when to escalate, and how to balance professional duty with self-protection. This suggests that observational learning is central to the development of ethical coping in emergency settings, particularly for less experienced nurses. Simulation, case reflection, and guided experience-sharing may help transform indirect exposure into constructive efficacy beliefs rather than fear-based avoidance.

Overall, the findings extend self-efficacy theory into the context of moral distress in emergency nursing by showing that efficacy beliefs are dynamically shaped by ethical outcomes, workplace relationships, embodied emotional responses, and indirect trauma exposure. Practically, interventions should move beyond individual stress management and incorporate ethics education, post-event debriefing, mentorship, and organizational support mechanisms to strengthen nurses' moral coping capacity.

### 4.1 Strengths of the study

This qualitative study fills a gap in the existing literature by deeply exploring the subjective coping mechanisms of ED nurses through the lens of Bandura's self-efficacy theory. Several methodological features strengthen the credibility of the findings. First, the use of an *a priori* theoretical framework (Bandura's four sources of self-efficacy) enhanced the analytical depth and provided a coherent lens for interpreting otherwise heterogeneous accounts of moral distress. Second, the application of maximum variation purposive sampling across gender, age, working years, and professional title (Table 2) maximized the range of perspectives represented within a single ED setting. Third, methodological rigor was supported through independent double coding, peer debriefing with a senior qualitative researcher, an explicit audit trail, and reflexivity statements documenting the coders' professional backgrounds and potential preconceptions. Finally, the integration of frequency information with thick descriptions and illustrative quotations provides a transparent view of both the salience and the lived texture of each subtheme, while explicitly avoiding inferential claims about prevalence.

### 4.2 Practical implications

The findings translate into multilevel, actionable recommendations with clearly identified implementers. At the unit level, head nurses should organize short, structured post-shift micro-reflection sessions (10–15 min) after ethically difficult events, supported by senior nurses and experienced mentors who can facilitate guided sharing and normalize emotional responses, a strategy proven effective in reducing the accumulation of moral residue and enhancing self-efficacy (17, 18). At the departmental and nursing administration level, nursing departments should develop and maintain an ED-specific ethical case library and integrate ethics-oriented simulation training (e.g., futile resuscitation, triage under scarcity, family-driven decision conflicts, workplace violence) into routine continuing education for ED nurses, particularly junior staff. At the hospital management level, leadership should establish standardized protocols for workplace violence prevention and response, accessible psychological support services, and explicit institutional safeguards (e.g., complaint-protection procedures and transparent ethical-consultation pathways) so that nurses do not experience defensive practice as the only available coping route. Finally, at the interdisciplinary level, ethics committees and multidisciplinary teams (physicians and clinical psychologists) should be involved in complex ethical case reviews and bedside ethical consultation, providing ED nurses with verbal persuasion, recognition and vicarious learning opportunities that strengthen self-efficacy from multiple sources.

### 4.3 Limitations

This study presents certain limitations that should be acknowledged. First, recruiting exclusively from the emergency department of a single tertiary general hospital in Guangdong Province restricts the transferability of the findings. The institutional culture, ethical climate, and clinical workflow described herein reflect a specific regional and organizational context; therefore, the application of these findings to other regions, hospital tiers, or healthcare systems should be assessed cautiously. Second, the qualitative design meant that data collection depended on self-reporting, making the thematic extraction susceptible to the researchers' subjective lenses. Furthermore, because ethical dilemmas are highly sensitive, social desirability bias may have influenced the interviews, leading some participants to mask feelings that deviate from professional expectations. Despite rigorous efforts to control for this bias—including strict confidentiality protocols and neutral questioning—its potential influence on data authenticity remains. Third, formal individual member checking of transcripts and final themes was not performed because of participants' rotating shift patterns and clinical workload; although key interpretations were summarized back to participants at the end of each interview, the absence of structured member checking may have limited opportunities to refine analytic interpretations. Finally, the frequencies and proportions presented in Table 3 reflect only spontaneous mentions of subthemes during open-ended interviews and should not be interpreted as estimates of prevalence; they are reported solely to indicate the relative salience of subthemes within this dataset. Building on these findings, subsequent research would benefit from multi-center, large-sample quantitative or mixed-methods approaches to verify the applicability and prevalence of these themes across diverse healthcare contexts.

## Data Availability

The original contributions presented in the study are included in the article/Supplementary material, further inquiries can be directed to the corresponding author.
